# Consistency and Sensitivity Evaluation of the Saudi Arabia Mental Health Surveillance System (MHSS): Hypothesis Generation and Testing

**DOI:** 10.2196/23965

**Published:** 2022-02-03

**Authors:** Nora A Althumiri, Mada H Basyouni, Nasser F BinDhim

**Affiliations:** 1 Sharik Association for Research and Studies Riyadh Saudi Arabia; 2 Decision Support for Research and Studies (IDM) Riyadh Saudi Arabia; 3 Kingdom of Saudi Arabia Ministry of Health Riyadh Saudi Arabia; 4 College of Medicine Alfaisal University Riyadh Saudi Arabia

**Keywords:** mental health, evaluation, screening, surveillance, data quality, surveillance system quality, surveillance system evaluation

## Abstract

**Background:**

Public health surveillance systems should be evaluated periodically, and the evaluation should include recommendations for improving the system’s quality and efficiency. Each surveillance system may have a unique situation in which evaluating its quality depends on its methodology, aims, and other factors, such as the frequency of repeating the survey in the case of survey-based surveillance.

**Objective:**

As the consistency of the surveillance system to capture demographic data and its sensitivity to monitor the intended health-related event are important indicators of the quality of the surveillance system, the aim of this article is to evaluate the Saudi Arabia Mental Health Surveillance System (MHSS) in terms of consistency and sensitivity via the scientific hypothesis testing process.

**Methods:**

The quality of the MHSS was assessed by examining (1) the consistency of the main demographic variables and (2) the sensitivity to changes in score between the 2 mental health screening tools used in the MHSS and between the 3 waves collected in 3 consecutive months. The assessment uses all data collected via the MHSS between May 2020 and July 2020. The first null hypothesis predicted there were differences between the distributions of the demographic variables between the 3 waves. The second predicted there were no differences between the scores of the Patient Health Questionnaire 9 (PHQ-9) and the Generalized Anxiety Disorder 7-item scale (GAD-7) between the 3 waves.

**Results:**

In terms of sampling variables (age, gender, and region), there were no significant differences between the 3 waves in age, using one-way ANOVA, nor in gender and region, using the chi-square test. In addition, there were no significant differences between the 3 waves in all other demographic variables, except in the income variable. However, in terms of the PHQ-9 score, the one-way ANOVA (*F*_2,12334_=8.05; *P*<.001) showed significant differences between waves. Similarly, significant differences between waves were found in the GAD-7 score (*F*_2,12334_=7.09; *P*=.001).

**Conclusions:**

The MHSS showed a consistent distribution of the sample demographic variables, while being sensitive to the changes in mental health scores across waves. The MHSS can generate an acceptable level of consistency and sensitivity to monitor mental health trends.

**International Registered Report Identifier (IRRID):**

RR2-10.2196/23748

## Introduction

Public health surveillance is the ongoing, systematic collection, analysis, interpretation, and dissemination of health data essential to the planning, implementation, and evaluation of public health programs for use in public health action to reduce morbidity and mortality and to improve health [1,2]. A surveillance system, however, is a collection of processes and components that enable a successful surveillance process, including data collection, data quality monitoring, data management, data analysis, interpretation of analytical results, information dissemination, and application of the information to public health programs [2]. Methodologically, the most popular surveillance systems in public health repeat cross-sectional surveys on a regular basis in the form of waves, which allow the data to be clustered by periods. Eventually, public health surveillance systems should generate information to inform decision-makers in many areas, including prevention program planning and management, health promotion, quality improvement, and resource allocation [2].

Mental disorders account for more collective disability burden than any other group of illnesses, including cancer and heart disease [3]. Disability can be caused by the effect of mental illness on emotions, thoughts, and daily function and the link between mental illness and general health, especially chronic diseases [4]. Historically, surveillance focused on infectious diseases, then broadened to other areas, such as chronic diseases [5]. Currently, mental health is increasingly recognized as a field in public health surveillance [5-7]. Mental health screenings are now included in established health surveillance surveys, such as the US Centers for Disease Control and Prevention’s (CDC) National Health Interview Survey (NHIS), National Health and Nutrition Examination Survey (NHANES), Behavioral Risk Factor Surveillance System (BRFSS), and the Substance Abuse and Mental Health Services Administration’s (SAMHSA) National Survey on Drug Use and Health (NSDUH) [5].

In April 2020, the Sharik Association for Research and Studies, formerly known as the Sharik Association for Health Research, established the Saudi Arabia Mental Health Surveillance System (MHSS) in collaboration with the Saudi Health Council (SHC), which is the highest national authority in the health domain in Saudi Arabia. The MHSS is a monthly phone-based cross-sectional survey conducted across the 13 administrative regions of Saudi Arabia, with a monthly sample of approximately 4000 participants. At the time of writing this manuscript, 3 waves have been successfully completed with a sample size of more than 12,000 participants. The full details of the MHSS’s scientific approach were published as a protocol paper [8]. The MHSS disseminates its results in an electronic dashboard developed for this project to inform decision-makers of the results as soon as possible and to compare the results with previous waves. A subcommittee under the SHC governs the MHSS, with members representing the main stakeholders across the SHC, including the Ministry of Health.

Although the need for public health surveillance systems has long been recognized, there is increasing pressure to improve the effectiveness of these surveillance systems via appropriate evaluation [9]. According to the CDC Guidelines for Evaluating Public Health Surveillance Systems, evaluation ensures that problems of public health importance are being monitored efficiently and effectively [1]. A recent systematic review focusing on existing approaches to surveillance system evaluation found only 10 originated from the public health surveillance field [9]. However, most of the approaches (13/15) could be defined as either frameworks or guidelines, as they provided a general or structured roadmap for the evaluation process, while fewer provided systematic information about how the evaluation should be carried out and, therefore, could be defined as methods [9]. However, all the assessment methods shared some common aspects and none suggested that all of their attributes would be relevant to each evaluation; instead, they could be selected according to the context and objectives of the evaluation at hand [1,9]. In terms of data quality evaluation, most guidelines suggest “data quality reflects the completeness and validity of the data recorded in the surveillance system” [1]. The first issue is that, with the advances of electronic data collection systems, the completeness of data is no longer an issue, as the data are always complete when an electronic system enforces it, which is the case with the MHSS. The second issue is that all of the guidelines focus on clinical data collection, although none focus on cross-sectional survey–based surveillance. Nevertheless, each surveillance system could have a unique approach and parameters for evaluating data quality depending on its methodology, aims, and other factors, such as the frequency of repeating the survey in the case of survey-based surveillance. Nevertheless, to ensure the quality of the MHSS, we considered all relevant attributes by evaluating the public health surveillance systems issued by the CDC [1].

Looking outside the public health domain for a more practical guide to assess surveillance system quality in general, the US Environmental Protection Agency (EPA) has published several versions of the Guidance for Data Quality Assessment: Practical Methods for Data Analysis, which is described as a “toolbox” of useful techniques for assessing the quality of data [10]. This guidance encourages the use of assumption and hypothesis testing to assess data quality and includes a well-detailed process for doing so [10].

In spite of that, public health surveillance systems should be evaluated periodically, and the evaluation should include recommendations for improving the system’s quality and efficiency [1,11]. As the consistency of the surveillance system in capturing demographic data and its sensitivity to monitor the intended health-related event are important indicators of the quality of the surveillance system, the aim of this article is to evaluate the MHSS in terms of consistency and sensitivity via the scientific hypothesis testing process by following the EPA Guidance for Data Quality Assessment.

## Methods

This study assesses the quality of the MHSS by examining (1) the consistency of the main demographic variables and (2) the changes between the scores of the 2 mental health screening tools used in the MHSS and between the 3 waves collected in 3 consecutive months.

### Null Hypotheses

With the assumption that the 3 waves were conducted in 3 consecutive months using the same data collection process and sampling methodology, which is further controlled by an automated sampling system [12], and the assumption that major demographic variables (eg, age, gender, education level, and marital status) will not change significantly over a short period of time, there will be no significant difference between the distribution of the demographic variables between the 3 waves. Thus, the first null hypothesis for this study is as follows: there are differences between the distributions of the demographic variables between the 3 waves.

With the assumption that the screening tools, the Patient Health Questionnaire 9 (PHQ-9) [13,14] and Generalized Anxiety Disorder 7-item scale (GAD-7) [15], are very sensitive to detecting changes in depressive and anxiety symptoms and that depressive and anxiety symptoms could vary significantly between individuals and within the same individual over a short period of time, as both tools measure symptoms within the prior 2 weeks, and because we are assessing different groups of individuals in each wave who will produce different scores, we assume that there will be significant changes in both scores across the 3 waves. Thus, the second null hypothesis for this study will be as follows: there is no difference between the scores of PHQ-9 and GAD-7 between the 3 waves.

In addition, internal consistency and test-retest reliability for both the PHQ-9 and GAD-7 were performed with the assumption that the system will generate an acceptable level of internal consistency and test-retest reliability.

### Variables

For the first experiment, the demographic variables to be tested are age, gender, region, education level, income level, marital status, and work status. Age, gender, and region are part of the sampling variables and are used to determine the completion of the sampling quota. Therefore, they will generate evidence about the quality of the sampling system because they are expected to be proportional according to the MHSS methodology [8].

For the second and third experiments, the total score of the PHQ-9 and GAD-7 will be used.

### Data

This study will use all the data generated by the MHSS between May 2020 to July 2020, which includes 12,337 participants (4004 participants in wave 1, 4180 participants in wave 2, and 4153 participants in wave 3). Each of the participants participated in one wave only. For the test-retest reliability, only 22 participants, 11 of whom (50%) were female, were interviewed twice in a pilot study before initiating the surveillance system, with one week between the two interviews.

### Data Analysis

For continuous variables (age and PHQ-9 and GAD-7 scores), a one-way ANOVA test will be used; box plots were also included to show the overall trend of the data. For other categorical variables, the chi-square test will be used. Internal consistency was assessed using the Cronbach α coefﬁcient, and the test-retest reliability was assessed with the intraclass correlation coefﬁcient. We used the SPSS statistical software, version 20 (IBM Corp).

## Results

### Assumption 1: Consistency of the Main Demographic Variables Between the 3 Waves Collected in 3 Consecutive Months

In terms of sampling variables (age, gender, and region), there were no significant differences between the 3 waves in age (*F*_2,12334_=0.71; *P*=.49), in gender (χ^2^_2_=0.1; *P*=.97), and in regions (χ^2^_24_= 4.8; *P*>.99).

In terms of other demographic variables, as shown in Table 1, there were no significant differences, except for income level.

**Table 1:** Participant demographics.

Characteristic			Wave 1, n=4004		Wave 2, n=4180		Wave 3, n=4153		All waves, N=12,337		*P* value
**Gender, n (%)**											
	Male	1989 (49.7)		2083 (49.8)		2058 (49.6)		6130 (49.7)		.97	
	Female	2015 (50.3)		2097 (50.2)		2095 (50.4)		6207 (50.3)			
**Education level, n (%)**											
	High school or less	1444 (36.1)		1457 (34.9)		1465 (35.3)		4366 (35.4)		.27	
	Undergraduate diploma	484 (12.1)		456 (10.9)		466 (11.2)		1406 (11.4)			
	Bachelor’s degree	1846 (46.1)		2022 (48.4)		1955 (47.1)		5823 (47.2)			
	Postgraduate degree (eg, master’s/PhD)	230 (5.7)		245 (5.9)		267 (6.4)		742 (6)			
**Income level, n (%)**											
	Less than 5000 SAR^a^ (<US $1331.47)	629 (15.7)		604 (14.4)		689 (16.6)		1922 (15.6)		<.001	
	Between 5001 to 8000 SAR (US $1331.74 to $2130.36)	687 (17.2)		595 (14.2)		668 (16.1)		1950 (15.8)			
	Between 8001 to 11,000 SAR (US $2130.62 to $2929.24)	619 (15.5)		610 (14.6)		664 (16)		1893 (15.3)			
	Between 11,001 to 13,000 SAR (US $2929.51 to $3461.83)	486 (12.1)		551 (13.2)		502 (12.1)		1539 (12.5)			
	Between 13,001 to 16,000 SAR (US $3462.10 to $4260.71)	542 (13.5)		628 (15)		559 (13.5)		1729 (14)			
	More than 16,000 SAR (>US $4260.71)	1041 (26)		1192 (28.5)		1071 (25.8)		3304 (26.8)			
**Region, n (%)**											
	Asir	321 (8)		322 (7.7)		321 (7.7)		964 (7.8)		>.99	
	Baha	316 (7.9)		311 (7.4)		314 (7.6)		941 (7.6)			
	Eastern Region	314 (7.8)		322 (7.7)		323 (7.8)		959 (7.8)			
	Hail	293 (7.3)		326 (7.8)		320 (7.7)		939 (7.6)			
	Jazan	312 (7.8)		321 (7.7)		324 (7.8)		957 (7.8)			
	Al Jouf	288 (7.2)		318 (7.6)		320 (7.7)		926 (7.5)			
	Madinah	321 (8)		325 (7.8)		316 (7.6)		962 (7.8)			
	Makkah	325 (8.1)		325 (7.8)		323 (7.8)		973 (7.9)			
	Najran	303 (7.6)		322 (7.7)		321 (7.7)		946 (7.7)			
	Northern Border	318 (7.9)		318 (7.6)		321 (7.7)		957 (7.8)			
	Qassim	309 (7.7)		328 (7.8)		320 (7.7)		957 (7.8)			
	Riyadh	301 (7.5)		323 (7.7)		320 (7.7)		944 (7.7)			
	Tabuk	382 (7.1)		319 (7.6)		310 (7.5)		912 (7.4)			
**Marital status, n (%)**											
	Never married	1548 (38.7)		1641 (39.3)		1611 (38.8)		4800 (38.9)		.77	
	Married	2196 (54.8)		2269 (54.3)		2279 (54.9)		6744 (54.7)			
	Divorced/separated	169 (4.2)		165 (3.9)		152 (2.7)		486 (3.9)			
	Widowed	91 (2.3)		105 (2.5)		111 (2.7)		307 (2.5)			
**Employment status, n (%)**											
	Employed	1579 (39.4)		1723 (41.2)		1638 (39.4)		4940 (40.0)		.29	
	Self-employed	179 (4.5)		189 (4.5)		170 (4.1)		538 (4.4)			
	Unemployed	1121 (28)		1081 (25.9)		1117 (26.9)		3319 (26.9)			
	Student	816 (20.4)		853 (20.4)		907 (21.8)		2576 (20.9)			
	Retired	309 (7.7)		334 (8)		321 (7.7)		964 (7.8)			

^a^1 SAR=US $0.27.

### Assumption 2: The Changes Between the 2 Mental Health Screening Tools Used in the MHSS and Between the 3 Waves Collected in 3 Consecutive Months

**Figure 1:**
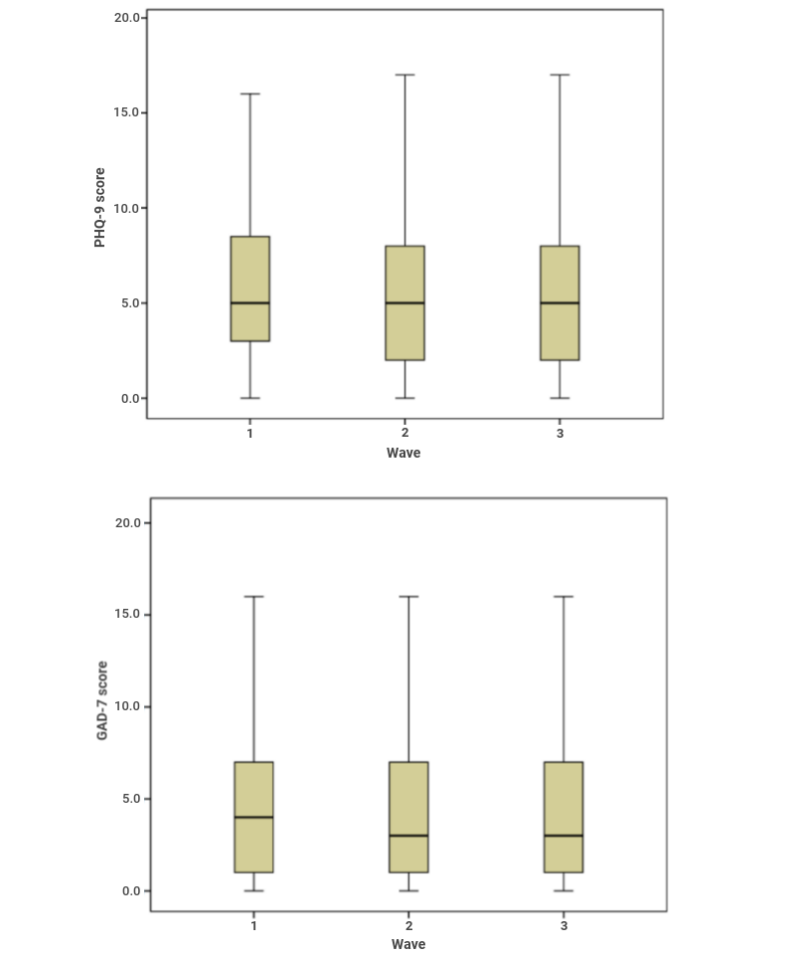
Box plots of the Patient Health Questionnaire 9 (PHQ-9) and Generalized Anxiety Disorder 7-item scale (GAD-7) across the 3 waves of data collection.

In terms of the PHQ-9 score, the one-way ANOVA (*F*_2,12334_=8.05; *P*<.001) showed significant differences between waves. Similarly, significant differences between waves were found for the GAD-7 score (*F*_2,12334_=7.09; *P*=.001). Figure 1 shows the box plots for both PHQ-9 and GAD-7.

### Assumption 3: Internal Consistency and Test-Retest Reliability

Internal consistency measures for both scales (α=.838 for the PHQ-9, α=.881 for the GAD-7) were good. In the analysis of test-retest reliability, the intraclass correlation coefﬁcient was 0.941.

## Discussion

### Principal Findings

This study has used assumption and hypothesis testing to evaluate the quality of the MHSS, which uses repeated cross-sectional surveys conducted in a systematic process as a surveillance system. The results showed that, for the first assumption, the null hypothesis was rejected for 6 out of 7 variables. This finding confirms the assumption that there will be no significant differences between the 3 consecutive waves in the main demographic variables if the surveillance system can generate high-quality data. Similarly, for the second assumption, the null hypothesis was rejected. This finding confirms the assumption that there will be significant differences in PHQ-9 and GAD-7 scores between the 3 consecutive waves if the surveillance system can generate high-quality data. In addition, the internal consistency and test-retest reliability showed an acceptable level of data consistency and reliability.

Although income level showed a significant difference on the chi-square test, a closer look reveals that the within-wave variability is still small, with the largest variability at 3% between the highest and lowest proportions. The age, gender, and region variables were used by the electronic data collection system to control the sample, to provide proportional distribution in gender and region and a similar average age as the general adult population in Saudi Arabia. The lack of differences in these 3 variables confirms the quality of the electronic sampling system and the assumption that it will generate the sample as planned.

The results showed that the MHSS generated a consistent composition of demographic variables in each wave, while showing sensitivity to the changes in the mental health screening measures (PHQ-9 and GAD-7). This finding provides an acceptable level of confidence that the MHSS is measuring what it is intended to measure. However, it is important to clarify that the PHQ-9 and GAD-7 have shown a high level of sensitivity and specificity across the literature, and that is part of the reason we selected them for mental health screening in this mental health surveillance system, to build on the strength of these well-established screening tools [13-15]. We acknowledge that it is hard to separate the effect of the screening tools from the surveillance system sensitivity; however, knowing that these two screening tools are sensitive to change and knowing that the surveillance system can generate consistent demographic segments each wave, we assumed that the change in PHQ-9 and GAD-7 scores will be significant if the surveillance system is functioning well overall. Confirming this assumption via hypothesis-based testing is not a solid confirmation of the sensitivity and consistency of the system, but it can generate some confidence until further evidence can be found.

This study used assumption and hypothesis testing to investigate the ability of the MHSS to generate an acceptable level of data consistency and sensitivity to monitor mental health trends, as defined by the EPA Guidance for Data Quality Assessment, although hypothesis-based evaluation is not an EPA or environmental research specific method. However, the accuracy of the results depends on the validity of the assumptions, which is a limitation for hypothesis testing in general. Another limitation is that the results came from internal comparison data from the same surveillance system, not from external data generated by another surveillance system or a cross-sectional survey using the same or similar methodology to provide external validity. Furthermore, although the ANOVA results used to test the second assumption show statistical significance, they might not be practically significant, especially with the short time period between waves. Finally, this study looked at the surveillance system quality as a broader concept than just data completion or data accuracy when comparing the same record to another record using the same participants. It showed that surveillance system quality could be investigated independently with various scientific testing methods.

### Conclusion

The MHSS showed a consistent distribution of the sample demographics, while being sensitive to the changes in mental health scores across waves. The MHSS can generate an acceptable level of consistency and sensitivity to monitor mental health trends.

### Data Availability Statement

Data will be available upon request via the Saudi National Health Research Center through NFB.

### Funding

This project is funded by the KACST (Grant 5-20-01-000-0001). The funder had no role in data collection, data analysis, data interpretation, or writing of the report. NFB had full access to all study data and had final responsibility for the decision to submit this research for publication.

## Abbreviations

BRFSS: Behavioral Risk Factor Surveillance System
CDC: US Centers for Disease Control and Prevention
EPA: US Environmental Protection Agency
GAD-7: Generalized Anxiety Disorder 7-item scale
KACST: King Abdulaziz City for Science and Technology
MHSS: Saudi Arabia Mental Health Surveillance System
NHANES: National Health and Nutrition Examination Survey
NHIS: National Health Interview Survey
NSDUH: National Survey on Drug Use and Health
PHQ-9: Patient Health Questionnaire 9
SAMHSA: Substance Abuse and Mental Health Services Administration
SHC: Saudi Health Council
